# Antioxidant Activity of Frozen and Freeze-Dried Drone Brood Homogenate Regarding the Stage of Larval Development

**DOI:** 10.3390/antiox10050639

**Published:** 2021-04-22

**Authors:** Ewelina Sidor, Michał Miłek, Monika Tomczyk, Małgorzata Dżugan

**Affiliations:** Department of Chemistry and Food Toxicology, Institute of Food Technology and Nutrition, University of Rzeszów, Ćwiklińskiej 1a St., 35-601 Rzeszów, Poland; mmilek@ur.edu.pl (M.M.); mwesolowska@ur.edu.pl (M.T.); mdzugan@ur.edu.pl (M.D.)

**Keywords:** drone brood, antioxidant activity, HPTLC, polyphenols, the stage of development

## Abstract

Drone brood is a little-known and poorly studied bee product used and valued in the treatment of many diseases, including male infertility and women’s menopausal disorders. The aim of this study was to evaluate the antioxidant activity of drone brood depending on the stage of larval development and the method of preservation. Aqueous and ethanolic homogenate extracts of drone brood were assayed for antioxidant activity (with the DPPH, FRAP, and ABTS methods), polyphenol, and flavonoid content. The extracts’ polyphenolic profiles were compared by the HPTLC method. Drone brood has been shown to be more active in the earlier stages of development (between days 7–11), with a decline in antioxidant activity in the later period (by the 14th day). The freeze-drying process did not cause significant changes in the antioxidant activity of brood preparations converted to dry mass. Based on the higher activity of the aqueous compared to 70% ethanolic extracts, it was shown that the dominant fraction of brood consisted of hydrophilic antioxidants. The results obtained with different methods were highly correlated, excluding those from the ABTS assay. The HPTLC method showed that the polyphenol fraction of drone brood homogenate consisted mainly of phenolic acids and flavonoids. It was shown that drone brood has valuable antioxidant properties that can be compared with royal jelly.

## 1. Introduction

Drone brood homogenate, less commonly called drone milk, is a little-known bee product that is often disregarded in articles relating to beekeeping products [1,2,3] or marginalized with a brief mention [4]. This bee product is obtained from drone larvae collected from the bee family in the spring or early summer, according to the authors of [5,6]. Although the main role of drones is to participate in the mating flight during which the queen is inseminated, a certain percentage of drones (5–10% of the adult bee colony population) is present in the hive throughout the beekeeping season [6]. Raw drone brood homogenate obtained by mechanical homogenization of drone larvae is a milky substance with a creamy consistency, ranging in color from white or yellow to green [5]. It is one of the bee products not widely known in Europe (except Romania and Russia) but recognized and frequently used as a natural medicine in some countries in the world, such as China, Zambia, Senegal, and Ecuador [6].

Due to its high levels of protein, vitamins, and hormones, drone brood is a natural remedy for men’s fertility problems, androgen deficiency during menopause in women, and prophylactic measures against conditions associated with osteoporosis. Additionally, it is recommended against nervous and mental diseases and thyroid disorders, has hepatoprotective and anti-atherosclerotic properties, and stimulates the immune system [5,6,7].

To obtain drone milk, male larvae must be taken on the tenth day after eggs have been laid, or on the seventh day in the larval stage. At this time, a drone larva weighs approximately 250–300 mg and contains all of the necessary substances and elements in high concentrations [6]. However, data on the most favorable age for larvae are inconclusive, and other authors have suggested using larvae in the later stages, i.e., 9–11 days old [8]. Native homogenate of drone brood obtained from drone cells is unstable during storage and requires additional stabilization to maintain the biological activity of its substances. Different methods are used to stabilize the biological activity of drone homogenate preparations, for example, mixing with honey, freezing, or adsorbing on lactose, but the most effective method is lyophilization, which allows one to preserve the active biological substances in drone brood for a long time [9]. Nowadays, the purest and most popular form of drone brood is the white-yellow powder obtained by freeze-drying fresh drone brood; it is available on the market under the name Apilarnil [6].

The chemical composition of drone brood homogenate is very diverse; it contains proteins (10–20%), carbohydrates (up to 5.0%), fats (5.0–6.3%), amino acids (11.4%), enzymes, hormones, and vitamins [5,7]. The complex composition of drone brood determines the number of pharmacological characteristics of this bee product, and in particular, the presence of antioxidant, immunotropic, adaptogenic, and anabolic actions [10]. In terms of the variety of vitamin content, drone brood homogenate ranks first among all beekeeping products, ahead of even royal jelly [11].

Drone brood is often referred to as the male equivalent of royal jelly due to its very similar chemical composition. Royal jelly is a substance secreted by the hypopharyngeal and mandibular glands of worker honeybees [1,3]. On average, royal jelly contains about 60% water, 12% sugar, 12% protein, 6% lipids, and 5% organic acids, sex hormones, minerals, and vitamins (C, D, E, B), which makes it a valuable bioactive product [3,12,13,14]. The antioxidant potential of royal jelly is attributed to its polyphenols content, mainly flavonoids [4,9,15,16,17,18,19,20]. Using a DPPH radical scavenging capacity assay, the researchers confirmed the antioxidant capacity of royal jelly at levels in the range of 30–80% of radical residue [14,17,18]. Antioxidant capacity was determined using FRAP assays, resulting in 180–220 mM Fe/100 g [14,17], while total phenolics measured by a Folin-Ciocalteu assay amounted to 23.49 mg GAE/100 g, on average [17]. It is assumed that both bee products, drone brood and royal jelly, show similar biological activity, and thus, antioxidant properties [7]. However, the composition of drone milk has been poorly studied with respect to the protein, amino acids, lipids, hormones, vitamins, and bioelements that make it a good source of antioxidants [10,11,21]. Flavonoids and other phenolic compounds are important components that contribute to the ability of drone brood to reduce oxidative stress and prevent cardiovascular diseases [22], but still have not been recognized.

So far, preliminary studies of the chemical composition and biological activity of drone brood have been carried out and demonstrated its high content of nutrients and bioelements. However, research on the antioxidant activity of this bee product as well as the influence of various environmental and biological factors on the antioxidant properties of drone brood is still lacking. Thus, the aim of this study was to evaluate the effect of the stage of larval development and method of preservation on the antioxidant properties of drone brood homogenates.

## 2. Materials and Methods

### 2.1. Chemicals

The chemicals [2,2-diphenyl-1-picrylhydrazyl; 2′-azino-bis (3-ethylbenzothiazoline- 6-sulfonic acid); 2,4,6-Tris(2-pyridyl)-s-triazine)], reagents (Folin–Ciocalteu reagent), and standards (p-coumaric acid, apigenin-7 glucoside, ferulic acid, naringenin, chrysin, and quercetin) were obtained from Sigma Aldrich (Saint Louis, MO, USA), and buffer components (chloroform, ethyl acetate, and formic acid) were purchased from Avantor Performance Materials Poland SA (APM, Gliwice, Poland).

### 2.2. Drone Brood Homogenates

Drone brood (7-, 11-, and 14-day-old) samples were collected from three apiaries distant from each other by at least 50 km in the southeastern part of Poland (Podkarpackie Voivodeship) during May and June 2020. Drone brood (50 g of each sample) of the *Apis mellifera carnica* was selected manually from the one-half drone frame, immediately sealed in sterile containers, and transferred to the laboratory. Each sample was homogenized using a tissue homogenizer with 7 mm plastic Omni Tips TM (TH 02, Omni International, Kennesaw, GA, USA). One part of the material was frozen at −70 °C, and then freeze-dried (using Alpha 1–2, LD plus, Martin Christ Gefriertrocknungsanlagen GmbH, Osterode, Germany). Dehydration was carried out for 72 h by cooling the sample to −55 °C at a standard pressure of 0.1 bar. The second part of the material was frozen at −18 °C. Drone brood samples were used in the experiments in both forms: freeze-dried or frozen, both ground in a mortar before analysis.

### 2.3. Preparation of Drone Brood Extract

Half a gram of drone brood sample (freeze-dried powder or defrosted homogenate) was extracted with 5 mL of distilled water or 70% ethyl alcohol. The samples were homogenized with a tissue homogenizer (TH 02, Omni International, Kennesaw, GA, USA) for 2 min at medium speed (15,000 rpm). The extracts were then centrifuged for 20 min at 10,000 rpm at 4 °C using a refrigerated centrifuge (MPW-351R, Med. Instruments, Warsaw, Poland). The supernatants were collected and then filtered through a 0.45 µm nylon syringe filter and stored in a refrigerator at 4 °C until further analysis, but not longer than 3 days. Before antioxidant activity tests, the extracts were diluted 10-fold using a proper solvent.

### 2.4. Antioxidants Assay

#### 2.4.1. DPPH Test

The inhibition of DPPH (2,2-diphenyl-1-picrylhydrazyl) radicals was measured according to the method previously used in our laboratory for honey analysis [23] with slight modifications. Twenty microliters of drone brood diluted extract was mixed with 180 µL of DPPH radical methanolic solution (0.1 mM) and kept in the dark for 30 min. In the control sample, the extract was replaced by proper solvent. After incubation, the absorbance of the test and control samples were measured at 517 nm in a microplate reader (EPOCH 2 microplate spectrophotometer, BioTek Instruments Inc., Winooski, VT, USA). The reduction of DPPH radicals (AA%) was calculated using the following equation:

AA% = [(Ao − As)/Ao] × 100,

where AA% is antioxidant activity, Ao is the absorbance of the control, and As is the absorbance of the tested samples.

#### 2.4.2. FRAP Test

The ferric reducing antioxidant powder (FRAP) test was performed according to Dżugan et al. [23]. The FRAP reagent contained 2.5 mL of a 10 mM solution of 2,4,6-tripyridyltriazine (TPTZ) in 40 mM HCl, 2.5 mL of 20 mM FeCl_3,_ and 25 mL of 0.3 M acetate buffer (pH 3.6). To 20 µL of diluted extract, 180 µL of FRAP reagent was added, and after incubation at 37°C for 10 min, the absorbance of the reaction mixture was measured spectrophotometrically at 593 nm (EPOCH 2) against a blank. Results were expressed as mmol Trolox equivalents (TE) per 100 g of drone brood sample (mmol/100 g) from the calibration curve prepared for Trolox in the range of 5–60 nmol/mL (y = 0.152x, R^2^ = 0.999).

#### 2.4.3. ABTS Test

The antioxidant capacity was determined using the ABTS test (2,2′-azino-bis(3-ethylbenzothiazoline-6-sulfonic acid) according to the method described by Re et al. [24] with slight modifications. ABTS solution (19.5 mg ABTS in 7.00 mL of distilled water) was mixed with 3.3 mg of potassium persulfate and stored in the dark for 24 h. Before use, it was diluted with phosphate buffer (0.1M, pH = 7.4) to obtain an absorbance value at 734 nm of about 0.7. Then 20 µL of each diluted drone brood extract was mixed with 180 µL of diluted ABTS radical solution. After 6 min incubation in the dark, the absorbance of each sample was recorded at 734 nm in an EPOCH 2 microplate reader against phosphate buffer. Results were calculated using the following equation:

ABTS% = [Ao − As)/Ao] × 100,

where Ao is the absorbance of the control and As is the absorbance of the tested samples.

### 2.5. Total Phenolic Content (TPC) Determination

The total content of phenolic compounds was determined using the Folin-Ciocalteu reagent according to Dżugan et al. [23] with minor modifications. To 20 µL of diluted drone brood extract, 100 µL of 10% Folin-Ciocalteu reagent was added, followed by 80 µL of 7.5% (*w*/*v*) sodium carbonate solution. The samples were kept in the dark for 60 min and then the absorbance was measured against the blank at 760 nm using the EPOCH 2 microplate spectrophotometer. The total content of phenolic compounds was expressed in mg of gallic acid equivalents (GAE) per 100 g of brood sample (mg GAE/100 g). The results were calculated based on a calibration curve prepared for gallic acid in the range of 0–125 µg/mL (y = 0.336x, R^2^ = 0.9914).

### 2.6. Total Flavonoid Content (TFC) Determination

The total content of flavonoids in the extracts of drone brood was assessed using the method of Biju [25]. One hundred microliters of the undiluted extract was mixed with 100 µL 2% AlCl_3_ (in methanol). The reaction mixture was incubated for 10 min at room temperature until the completion of the reaction. The absorbance of the solution was then measured at 415 nm with an EPOCH 2 microplate reader against a methanol blank. The total content of flavonoids in the extracts of drone brood was expressed in mg of quercetin equivalent (QE) per 100 g of brood sample (mg QE/100 g). The results were calculated on the basis of a calibration curve prepared for quercetin in the range of 0–125 µg/mL (y = 0.0655x, R^2^ = 0.9999).

### 2.7. Identification of Antioxidant Compounds by HPTLC

Analyses of all obtained ethanolic extracts in 70% ethanol of the drone brood samples were performed on HPTLC Silica Gel 60 F_254_ plates (20 cm × 10 cm) purchased from Merck (Darmstadt, Germany). Forty microliters of each drone brood homogenate extract were applied to the plate as 11 mm bands from the lower edge of the plate at the rate of 200 nL/s using a semi-automated HPTLC application device (Linomat 5, CAMAG, Muttenz, Switzerland). The chromatographic separation was performed in a chromatographic tank saturated for 20 min with the mobile phase composed of chloroform: ethyl acetate: formic acid [5:4:1 v:v:v], and developed to a distance of 85 mm. The obtained results were documented using an HPTLC imaging device (TLC Visualizer, CAMAG, Muttenz, Switzerland) under white light, 254 and 366 nm. In addition, each plate was derivatized with 0.05% DPPH reagent (in methanol) using an automated TLC derivatizer (CAMAG Derivatizer, Muttenz, Switzerland). After derivatization, the plates were imaged under white light and 366 nm. The obtained chromatographic images were analyzed using HPTLC software (Vision CATS, CAMAG, Muttenz, Switzerland).

### 2.8. Statistical Analysis

All calculations were made in triplicate. For the obtained data, mean values and standard deviations were calculated. The correlations between the obtained parameters were analyzed by Pearson coefficient (r). Significant differences were calculated by two-way analysis of variance followed by NIR Fisher’s test of significant difference (*p* **<** 0.05). Multidirectional analysis of variance with the Wilks test was performed to determine the influence of independent factors as well as the interactions between them at the significance level *p* = 0.05. Principal component analysis (PCA) was used to find the relationship between the parameters tested in drone brood depending on the developmental stage and the method of fixation. All calculations were made using the Statistica 13.3 software (StatSoft, Tulsa, OK, USA).

## 3. Results

### 3.1. Antioxidant Activity (Measured by DPPH, ABTS, FRAP), Total Phenolic and Flavonoid Content of Frozen Drone Brood Homogenates

In the first step of the study, the antioxidant activity of extracts obtained from frozen drone brood samples was measured with the DPPH, ABTS, and FRAP methods. Moreover, total polyphenol and flavonoid content were analyzed. The comparison covered brood collected from three apiaries (I, II, III) at three different stages of larval development (day 7, 11, and 14). Additionally, the examination of brood homogenate extracts obtained with two solvents, water and 70% ethanol, was performed on the basis of earlier findings [6], where significant differences between these two extracts were noticed. The results are presented in Table 1.

Among tested aqueous extract samples, the highest antiradical activity (DPPH test) was found for the 11-day-old drone brood, in comparison to days 7 and 14 or apiaries I and II (*p* < 0.05). Any significant differences (*p* > 0.05) were observed for the 7- and 14- day-old drone brood aqueous extracts. Lower levels of antioxidant potential in 70% ethanol extracts were found, with the lowest for 14-day-old and highest for 7 -day-old drone brood.

The analysis of antioxidant potential measured with ABTS reagent showed an inverse correlation with the DPPH results (Table 1). The activity of homogenates extracted with 70% ethanol was found to be higher than that of the aqueous extracts, and an inverse relationship was observed for aqueous extracts, where the 11-day-old drone brood showed the lowest activity on average.

Minor differences between samples were observed for reducing potential (in FRAP test). When analyzing the mean values of homogenate activity, the same relationship as confirmed by the DPPH method was observed, where 11-day-old drone brood showed the strongest activity, while slightly lower activity was found with the 7- and 14-day-old samples (*p* < 0.05). The use of 70% ethanol as the extractant resulted in a lower ability to reduce Fe(III) ions by about 48% in all tested extracts, with the same tendency observed in all developmental stages.

High phenolic compound content was found in the aqueous-extract frozen drone brood. A statistically significant relationship (*p* < 0.05) was found between the developmental stage and the polyphenol content, regardless of the apiary origin of the sample. Similarly to the DPPH and FRAP methods, the mean content of polyphenols was the highest on day 11 of development, but decreased in the14-day-old brood. A similar relationship was observed for ethanolic extracts, where the content of phenolic compounds was 65–70% lower and differed significantly depending on the stage of development and apiary origin (*p* < 0.05).

For flavonoids as well, water proved to be a better extractant than 70% ethanol. The highest level was shown for 11-day-old drone brood regardless of apiary origin (*p* > 0.05). Moreover, average flavonoid content in brood on day 14 of development decreased by 38.8% compared to day 11 of development (*p* < 0.05).

### 3.2. Antioxidant Activity (Measured by DPPH, ABTS, FRAP), Total Phenolic and Flavonoid Content of Freeze-Dried Drone Brood Homogenates

In the second step of the study, the antioxidant activity (DPPH, ABTS, FRAP methods), as well as total polyphenol and flavonoid content, were analyzed in extracts of freeze-dried drone brood (Table 2). The comparison was arranged in the same manner as in Section 3.1. The highest antiradical activity measured with the DPPH method was observed in 11-day-old drone brood. The obtained value was significantly different (*p* < 0.05) from the other results, both in terms of apiary origin and developmental stage. The lowest activity was observed in 14-day-old brood collected from apiary III. The observed relationship was the same as demonstrated for frozen brood. Similarly, the use of 70% ethanol as an extractant resulted in a decrease in the antioxidant activity of the tested extracts, by 70%. In this case, significant changes in antioxidant activity depending on the apiary origin of brood were demonstrated (*p* < 0.05).

In lyophilized preparations, the results for antioxidant activity measured with the ABTS test were higher for 70% ethanolic extracts compared to aqueous counterparts. Moreover, regardless of the stage of maturity and extractant used, the high variability (*p* < 0.05) in antioxidant potential was observed to be dependent on the apiary origin of the samples.

The highest reducing power (FRAP test) was observed with 11-day-old drone brood extracted with water, while the lowest was observed with 14-day-old brood ethanolic extract. The use of ethanol as an extractant resulted in a decrease in reducing ability of extract, by an average of 73% for 7- and 11-day-old brood and 80% for 14-day-old brood. The differences in activity were significant between apiaries (*p* < 0.05) and developmental stages (*p* < 0.05).

Similarly to frozen brood, the lyophilizates were characterized by high phenolic compound content. The highest average content was observed with 11-day-old brood homogenate. The use of ethanol for extraction resulted in a decrease in content of the tested compound, on average by 65% for 7- and 14-day-old brood and 71% for 11-day-old brood.

The analysis of the flavonoid level in brood extracted with water and fixed by lyophilization showed the highest levels on average in 11-day-old brood samples, with the highest content in the brood obtained from apiary I. Brood origin significantly influenced flavonoid content (*p* < 0.05). What is more, the developmental stage significantly affected total flavonoid content (*p* < 0.05). The use of ethanol as an extractant resulted in a significant reduction in the content of the tested compounds in extracts, by 47 to 67%.

### 3.3. Loss of Bioactive Compounds and Antioxidant Activity during Freeze-Drying of Drone Brood Homogenate

**Figure 1:**
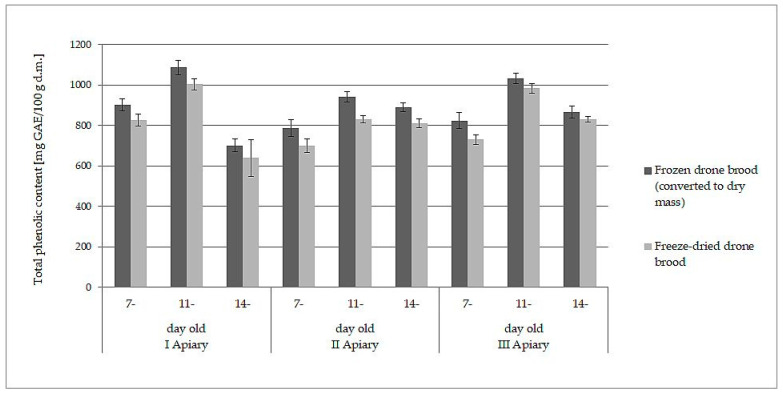
Comparison of total phenolic content (expressed as dry mass) in frozen and freeze-dried drone brood at different stages of development (7-, 11-, and 14-day-old larvae).

**Figure 2:**
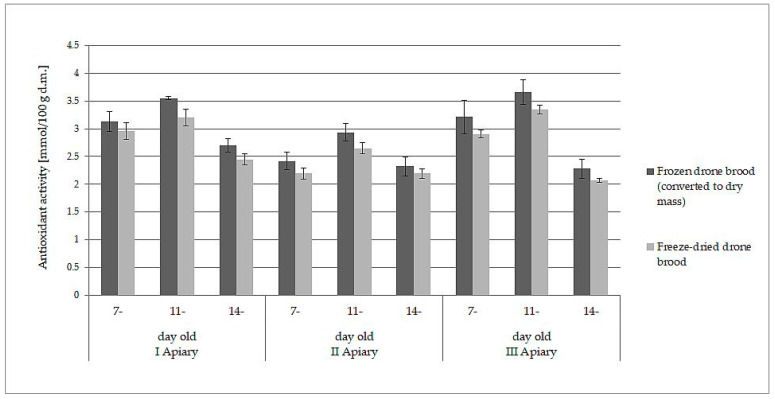
Comparison of antioxidant activity (FRAP method, expressed as dry mass) in frozen and freeze-dried drone brood at different stages of development (7-, 11-, and 14-day-old larvae).

The white-yellow powder obtained by lyophilization after reconstitution in water formed a white-yellow, milky suspension strongly resembling refrosted homogenate of drone brood. Moreover, both samples behaved analogously when they were extracted with water and ethanol. The data presented in Table 1 and Table 2 may lead directly to the wrong conclusion that frozen drone brood contains less antioxidants, phenolic compounds, and flavonoids. However, the differences in the content of the discussed compounds were due to different expressions of the data: wet mass for frozen drone brood and dry mass for freeze- dried drone brood. When the results for frozen homogenates were converted into dry mass (using the loss of water during freeze-drying), direct comparison to freeze-dried brood was then possible. A slight decrease in antioxidant compound activity (by 6–10%) and total phenolic compound content (by 7–11%) during the lyophilization process was observed (*p* > 0.05). Comparisons of results for TPC (Figure 1) and FRAP (Figure 2) are shown below.

### 3.4. Statistical Analysis

The results for the antioxidant activity of frozen brood showed strong positive correlations with the DPPH, FRAP, TPC, and TFC parameters (Table S1) measured by Pearson correlation coefficient (r = 0.838–0.929). Conversely, the ABTS results showed a strong negative correlation with all other tested parameters. A similar trend was observed for the freeze-dried bee product. The results of the DPPH, FRAP, and TPC, as well as TFC assays, showed a strong positive correlation (r = 0.814–0.929). In this case, a strong positive correlation for the ABTS and other results was observed, excluding the DPPH method (r = −0.828). A strong positive correlation was found between the results for frozen and freeze-dried brood, where correlation coefficients for all parameters were above 0.82 (Table S1).

**Figure 3:**
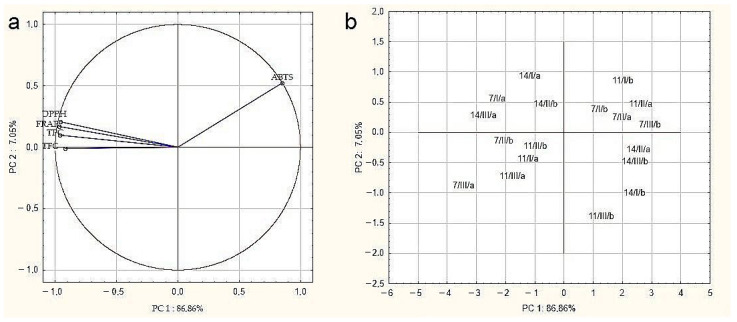
Principal component analysis (PCA)–(**a**) biplot of scores and loadings of data obtained for antioxidant activity, total phenolic compounds, and flavonoid content of tested drone brood samples; (**b**) the scatter plot of results in the space of the first two main components (7, 11, 14 days of development/I,II, III-apiary number/a, b- extractant: a- water, b- ethanol).

The relationships between the determined parameters for drone brood samples were verified by principal component analysis (PCA) (Figure 3). Variables displayed in Figure 3a confirmed the strongest positive correlation between the DPPH, FRAP, TPC, and TFC methods, demonstrated by close proximity. Conversely, a negative correlation was demonstrated between the antioxidant activity measured by ABTS and other assays, which were located in different areas of the biplot. Moreover, it can be noticed that all examined parameters had a significant impact on overall drone brood quality due to their location close to the projection circle. The analysis showed that the tested samples were characterized by high heterogeneity (Figure 3b), and it was not possible, based on the tested parameters, to clearly classify the samples in terms of geographical origin, developmental stage, or solvent used.

The results were also examined by multivariate significance test (Wilks test). The developmental stage and location of the apiary were found to have a significant influence on the values of tested parameters of biological activity. The interaction effect between the main factors was evidenced by the test probability level *(p* = 0.08) for the apiary number and developmental stage.

### 3.5. High-Performance Thin Layer Chromatography Polyphenolic Profile

High-performance thin-layer chromatography (HPTLC) recently has been applied in the profiling of phenolic compounds in various types of products. During HPTLC separation, polyphenols occur as bands of different intensity, which is crucial for the construction of characteristic fingerprints [26,27]. Using the CAMAG system, it is possible to visualize the polyphenolic profiles of multiple samples simultaneously under identical separation conditions. Such analyses have been intensively studied for use in quality assessments of bee products [28,29].

Drone brood has not been analyzed before in terms of its phenolic compound profile, but according to the literature, it is expected to show a composition similar to royal jelly [5,11]. Therefore, based on research relating to the analysis of the phenolic compound profile for royal jelly, the following standards were selected: *p*-coumaric acid, apigenin 7-glucoside (api 7-glu), ferulic acid, naringenin, chrysin, and quercetin [30]. Preliminary tests applying aqueous and ethanolic extracts to TLC plates yielded better band quality, were easier to perform, and required less time to apply the ethanol-extracted drone brood samples; a version of this approach was therefore selected for thin-layer chromatography analysis.

**Figure 4:**
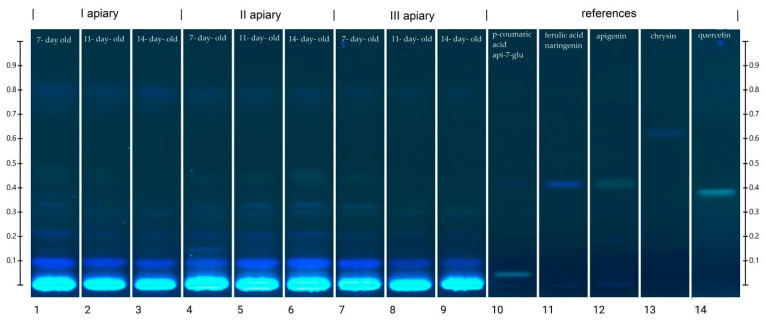
HPTLC chromatogram of phenolic compounds at 366 nm in frozen drone brood homogenates (1–9). Phenolic compound standards used: (10) *p*- coumaric acid, apigenin 7-glucoside (api 7-glu), (11) ferulic acid, naringenin, (13) chrysin, (14) quercetin.

The chromatograms showing the separation of phenolic acids and flavonoids in the frozen drone brood homogenates are demonstrated in Figure 4. Obtained profiles contained both blue (typical of phenolic acids) and yellow (characteristic of flavonoids) bands [31]. The identification of phenolic compounds was performed on the basis of R_f_ values and colors of the selected standard bands (Table S2). The HPTLC chromatograms indicated strong similarities in composition between the identified compounds and drone brood samples of different stages of development and apiary origin.

The location of the bands and their color intensity on the TLC chromatogram corresponds to qualitative and quantitative differences in the tested substance, respectively. The analysis of the chromatogram of the drone brood homogenate showed yellow and blue bands as dominant (Figure 4). In all tested extracts, the presence of phenolic acids with blue bands in the UV_366_ light was confirmed, and two major compounds with the retention coefficients R_f_ = 0.09 and R_f_ = 0.22 were observed (Table S3). The intensity of the bands was used to estimate the approximate content of a particular compound (Table S3). It was found that the 7-day-old brood, regardless of the apiary of origin, was the richest source of phenolic acids, while the poorest source was the 14-day-old. Moreover, all bands in the sample from apiary II identified as phenolic acids occurred at the highest intensity. In the drone brood, apigenin, one of the flavonoids, was also identified as a yellow band under UV_366_ light. The most intense color of this band was found in extracts from apiary II, for the 14-day-old brood, whereas the least visible apigenin bands were found for extracts from apiary I (Table S3). Moreover, flavonoids with the retention factor R_f_ = 0.30 (pale yellow), as well as two compounds with the retention factor R_f_ = 0.33 (pale blue) and R_f_ = 0.80 (blue) were detected in the studied extracts of drone brood. Due to the limited availability of reference substances and an unrecognized topic, no detailed identification of these compounds was possible.

**Figure 5:**
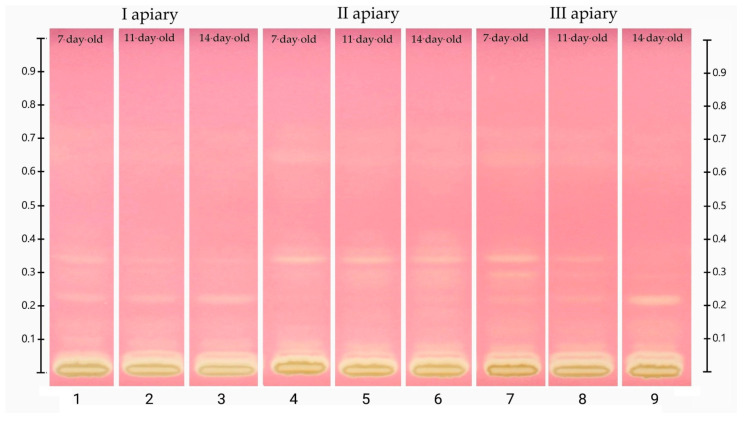
HPTLC chromatogram after derivatization with developing reagent (0.05% DPPH); (1–3 apiary I, 4–6 apiary II, 7–9 apiary III)**.**

The plate was developed with 0.05% methanolic 2.2-diphenylpicrylhydrazyl (DPPH) reagent, followed by drying for 2 min in a derivatization chamber (Figure 5). The obtained results were documented using an HPTLC imaging device. In this derivatization method, the plates show yellow bands on the violet background due to the discoloration of the DPPH reagent in reaction to components with antiradical properties. The most intense discoloration was observed for bands with an R_f_ coefficient of approx. 0.34 and approx. 0.22 (Table S3). The first of these compounds was dominant in samples from apiary II and the second, in samples from apiaries I and III, especially in the 14-day-old brood. Based on the comparison with the data in Figure 4, it can be concluded that these bands show a blue color at 366 nm and can be assigned to the group of phenolic acids. Among the tested polyphenol standards, naringenin, ferulic acid, and apigenin showed the strongest antiradical activity, manifested as intense yellow bands.

## 4. Discussion

Drone brood is a valuable bee product; however, it is strongly unstable after removal from the comb. Thus, fixing the brood by lyophilization is a modern but rarely used method that maintains the stability of the homogenate without reducing the quality of the tested material [32]. It was found that after re-dissolving in water, freeze-dried brood showed the same color and consistency as the raw homogenate. Moreover, both frozen and freeze-dried homogenates were observed to have low solubility in water. In turn, the extraction with ethanol was also problematic and resulted in the formation of a large amount of precipitate, possibly containing proteins. The low solubility of the homogenate in water and extraction of only low molecular antioxidants with ethanol may lead to analytical difficulties and non-reproducibility of results.

Both aqueous and ethanolic extracts of drone brood homogenates were examined with standard methods used in the antioxidant analysis of biological samples. The purpose of using two different solvents (water, 70% ethanol) was to diversify the hydrophilic and hydrophobic fractions of antioxidants present in the brood as well as to allow for comparison with data reported in the literature. It was found that the younger the age of the larvae tested, the higher the activity observed. The 11-day-old brood showed the highest antioxidant activity while the lowest was observed with the 14-day-old brood (Table 1 and Table 2). When comparing drone broods at the same stage of development, statistically significant differences were found to be related to the apiary of origin. The brood from apiary II showed the highest antioxidant activity, whereas that from apiary III had the lowest. Both apiaries were located closer to each other and did not differ from apiary I, which was located at a greater distance. This suggests that geographical origin is of less importance to a brood’s characteristics than hive conditions, as was earlier reported [33,34].

In terms of the antioxidant potential of drone brood, the obtained results were in agreement with other author findings. Silici [35] examined the bioactivity of apilarnil (7-day-old brood) and showed total phenolic content in the range of 760–940 mg GAE/100 g and DPPH values of aqueous extracts up to 81% under the same analytical conditions [35]. Although developmental changes in antioxidant potential of drone brood were demonstrated for the first time, a similar study on drone brood amino acids reported an increase in aspartic acid, glutamic acid, cysteine, and lysine content corresponding with the developmental phase of the drone brood; these increases, can also improve the activity of brood in alleviating oxidative stress [11,36,37]. Developmental changes in nutritional value for bee male larvae were studied only by Ghosh et al. [37], who analyzed, among other factors, the antioxidant properties of ethanolic extracts of Danish Buckfast honeybee drone larvae (fifth larval instars from the open-cell phase), pupae (brood with a dark eye), and adults (right after biting out of the comb). The cited authors reported antioxidant activity measured with the DPPH test for larvae and late pupae at levels of 6% and 26%, respectively, which can be compared to our results where the activity between 7- to 14-day-old larvae ranged from 11 to 14.5%. Moreover, the cited study [37] checked antioxidant activity with the ABTS reagent and found higher activity in ethanolic extracts (40% for adult drones). A similar relationship was observed in our study, where the activity for ethanolic extracts was higher than that observed for aqueous extracts and amounted to 52–65%.

The high biological activity of drone brood was confirmed by several reference methods, and the results were well-correlated, except for the ABTS method (Table S1). The aqueous extracts were more abundant in antioxidants compared to ethanolic ones regardless of the method used, and only in the ABTS test was an adverse relationship observed. In the case of aqueous extracts, the proteins (including enzymes), vitamins, and water-soluble polyphenols may be considered as antioxidant components. However, drone brood antioxidants are represented also by unsaturated fatty acids in free and bound form, including unique decenoic acids [7,38] and some hydrophobic phenolic compounds, which can be 15–30 times more active in scavenging free radicals [39]. These components probably influenced the antioxidant activity of ethanolic protein-free extracts.

Although the ABTS and DPPH tests can be comparable, and both are applied for the determination of lipophilic and hydrophilic antioxidants [40,41], a negative correlation between their results was observed. The adverse relation observed for water extracts can be explained by the following causes: (1) the acidic pH of aqueous extracts (pH 5.0), which can affect the ABTS results [32,33], (2) the greater efficiency of sulfhydryl groups of protein in scavenging of DPPH radicals [42,43], and (3) the possibility of a subsequent reaction of a product formed between the ABTS cation radical and parent compounds, which then reacts more quickly with ABTS [44]. The last cause seems to be the most probable, given that ethanolic extract contained many phenolic acids and flavonoids.

The obtained results indicated that the tested material is a rich source of flavonoids compared to honey, which contains about 5–8 mg/100 g of the tested compounds, but less than propolis, which contains about 379 mg/100 g of flavonoids [45].

As various polyphenols differ in their antioxidant effectiveness [46], the chromatographic profiles of the samples provided basic information about the antioxidant fraction composition and could be helpful as markers of developmental stage. In recent years, high-performance thin-layer chromatography has become an increasingly popular method of analyzing different compounds in bee products such as 10-hydroxy-2-decenoic acid [47], the sugar profile in honey [48], and the profile of phenolic compounds in bee pollen [49]. However, so far, this method has not been used for the analysis of drone brood. As the data on drone brood polyphenols was completely lacking and the composition of royal jelly and brood was presumed to be similar, data reported in the literature for polyphenol analysis of royal jelly were followed. Based on UHPLC-Orbitrap-MS analysis [30], the occurrence of naringenin, genistein, chrysin, apigenin 7-glucoside has been confirmed. However, using the HPTLC method, it was possible to identify only apigenin and confirm the presence of numerous phenolic acids. Probably, the used method was not sensitive enough to detect very low concentrations of other flavonoids. On the other hand, the lack of the above-mentioned compounds in drone brood homogenates may indicate that royal jelly and drone brood differ in their phenolic compound profiles. When the plate was derivatized with DPPH solution, an additional observation about the antiradical potential of separated compounds (bands) was made. Such derivatization of TLC plates is not commonly used. The most intense yellow band demonstrating the strongest DPPH radical reducing effect was observed with the ferulic acid, naringenin, and apigenin standards (Table S2). However, the applied apigenin glycoside did not show any effect, which was consistent with data in the literature suggesting that flavonoid glycosides usually have lower antioxidant activity than aglycons [50,51]. However, the proposed, new detection method (HPTLC-DPPH) allowed confirmation of the antiradical potential of drone brood homogenates.

According to the literature, all bee products are considered to be a potential source of natural antioxidants with order of decreasing activity from propolis to royal jelly, bee bread, pollen, and honey [2,3]. It was shown that drone brood, often considered as the male equivalent of royal jelly, exhibits strong antioxidant properties. Comparing data reported for royal jelly [1,17,18] with those obtained for drone brood in this study, it can be considered that the antioxidant potential of drone brood is more beneficial than royal jelly.

## 5. Conclusions

The study confirmed that the antioxidant potential of drone brood homogenate depends on the stage of larval development. An increase between 7 and 11 days followed by a reduction at later stages (14 days) was observed. The obtained results support the use of drone brood up to 10 days old, which is technologically beneficial due to the possibility of easily removing larvae from non-capped patch cells. The fixing of drone brood homogenate by the freeze-drying process did not cause significant changes in antioxidant activity when compared in terms of dry mass. Based on the comparison of water and ethanol extraction results, it was found that hydrophilic antioxidants form a major antioxidant fraction of drone brood; this was confirmed by several methods and supported by statistical analysis. The similarity of qualitative polyphenolic profiles of extracts investigated by the HPTLC method was determined, and the same phenolic acids and flavonoids were identified in drone brood ethanolic extracts regardless of the stage of larval development. Furthermore, the usefulness of the HPTLC method in the identification of polyphenols of drone brood homogenates was demonstrated. Drone brood has valuable antioxidant properties that can be successfully compared with royal jelly. The biological properties reported in the literature for drone brood homogenate can be attributed to its confirmed antioxidant activity. However, further study will be needed to identify the main active substances.

## Figures and Tables

**Table 1:** Antioxidant activity (measured with DPPH, ABTS, and FRAP methods) and total phenolic and flavonoid content of frozen drone brood homogenates extracted with water and 70% ethanol.

Developmental Stage	Extractant	Apiary I	Apiary II	Apiary III	Average
Antioxidant Activity DPPH [%] (*n* = 3) (±SD)					
7-day-old	water	14.3 ^b^ ± 0.1	16.4 ^c^ ± 1.1	11.9 ^a^ ± 0.5	14.2 ± 2.2
	ethanol 70%	5.3 ^DE^ ± 0.1	5.8 ^DE^ ± 0.4	1.7 ^B^ ± 0.1	4.3 ± 2.2
11-day-old	water	24.8 ^d^ ± 0.4	23.2 ^d^ ± 1.6	12.4 ^a^ ± 0.2	20.1 ± 6.7
	ethanol 70%	4.4 ^CDE^ ± 0.4	4.2 ^CD^± 1.2	3.3 ^BC^ ± 0.1	4.0 ± 0.6
14-day-old	water	15.8 ^bc^ ± 0.2	16.9 ^c^ ± 1.4	11.2 ^a^ ± 0.6	14.7 ± 3.0
	ethanol 70%	3.0 ^BC^ ± 0.1	6.0 ^E^ ± 0.9	0.00 ^A^	3.0 ± 3.0
Antioxidant Activity ABTS [%] (*n* = 3) (±SD)					
7-day-old	water	13.5 ^d^ ± 0.5	7.3 ^b^ ± 1.0	17.2 ^e^ ± 0.2	12.7 ± 4.9
	ethanol 70%	20.2 ^BC^ ± 0.7	21.4 ^C^ ± 2.6	19.4 ^B^ ± 0.6	20.4 ± 1.0
11-day-old	water	2.8 ^a^ ± 0.2	5.5 ^ab^ ± 0.2	10.5 ^c^ ± 0.4	6.3 ± 3.9
	ethanol 70%	24.9 ^D^ ± 0.5	21.5 ^C^ ± 3.1	16.4 ^A^ ± 1.6	21.0 ± 4.3
14-day-old	water	7.5 ^b^ ± 0.2	11.4 ^cd^ ± 1.0	15.8 ^e^ ± 0.5	11.6 ± 4.2
	ethanol 70%	24.3 ^D^ ± 2.4	21.4 ^C^ ± 2.1	16.2 ^A^ ± 1.0	20.7 ± 4.1
Ferric Reducing Antioxidant Power FRAP [mmol/100 g] (*n* = 3) (±SD)					
7-day-old	water	1.0 ^abcd^ ± 0.1	1.2 ^de^ ± 0.1	0.9 ^ab^ ± 0.1	1.0 ± 0.1
	ethanol 70%	0.4 ^A^ ± 0.1	0.6 ^A^ ± 0.1	0.5 ^A^ ± 0.0	0.5 ± 0.1
11-day-old	water	1.1 ^cde^ ± 0.0	0.9 ^a^ ± 0.1	1.3 ^e^ ± 0.1	1.1 ± 0.2
	ethanol 70%	0.6 ^A^ ± 0.0	0.5 ^A^ ± 0.1	0.5 ^A^ ± 0.0	0.5 ± 0.0
14-day-old	water	0.9 ^a^ ± 0.0	0.9 ^abc^ ± 0.1	1.1 ^bcde^ ± 0.2	0.9 ± 0.1
	ethanol 70%	0.5 ^A^ ± 0.1	0.5 ^A^ ± 0.9	0.4 ^A^ ± 0.1	0.5 ± 0.0
Total Phenolic Content TPC [mg GAE/100 g] (*n* = 3) (±SD)					
7-day-old	water	234.6 ^b^ ± 10.0	267.9 ^e^ ± 14.2	251.5 ^c^ ± 14.5	251.3 ± 16.6
	ethanol 70%	74.4 ^B^ ± 9.2	101.2 ^G^ ± 10.3	91.8 ^E^ ± 10.2	89.1 ± 13.6
11-day-old	water	282.7 ^f^ ± 12.6	320.4 ^h^ ± 9.3	256.0 ^d^ ± 10.5	289.4 ± 32.4
	ethanol 70%	69.9 ^A^ ± 7.2	95.2 ^F^ ± 9.1	80.4 ^D^ ± 9.0	81.8 ± 12.7
14-day-old	water	224.7 ^a^ ± 10.1	285.2 ^g^ ± 7.4	225.7 ^a^ ± 10.0	245.2 ± 34.7
	ethanol 70%	78.9 ^CD^ ± 10.3	104.7 ^H^ ± 12.2	77.4 ^C^ ± 10.1	87.0 ± 15.3
Total Flavonoid Content TFC [mg/100 g] (*n* = 3) (±SD)					
7-day-old	water	9.9 ^cde^ ± 1.2	6.7 ^b^ ± 0.2	9.5 ^cd^ ± 0.2	8.7 ± 1.7
	ethanol 70%	0.8 ^A^ ± 0.1	0.6 ^A^ ± 0.3	0.6 ^A^ ± 0.1	0.7 ± 0.1
11-day-old	water	10.9 ^de^ ± 0.7	10.3 ^cde^ ± 0.2	11.5 ^e^ ± 0.2	10.9 ± 0.6
	ethanol 70%	1.0 ^A^ ± 0.1	0.7 ^A^ ± 0.1	1.3 ^A^ ± 0.1	1.0 ± 0.3
14-day-old	water	9.0 ^c^ ± 1.0	4.1 ^a^ ± 0.2	6.8 ^b^ ± 0.1	6.7 ± 1.9
	ethanol 70%	1.5 ^A^ ± 0.1	0.4 ^A^ ± 0.1	1.2 ^A^ ± 0.1	1.0 ± 0.6

^a, b, c, d, e, f, g, h^—for aqueous extracts lowercase superscript letters indicate significant differences for data in columns (apiary number) and rows (developmental stage) obtained by two-way ANOVA (NIR Fisher test. *p* < 0.05). ^A, B, C, D, E, F—^for ethanolic extracts uppercase superscript letters indicate significant differences for data in columns (apiary number) and rows (developmental stage) obtained by two-way ANOVA (NIR Fisher test. *p* < 0.05). SD is the standard deviation.

**Table 2:** Antioxidant activity (measured with DPPH, ABTS, FRAP methods) and total phenolic and flavonoid content of freeze-dried drone brood homogenates extracted with water and 70% ethanol.

Developmental Stage	Extractant	Apiary I	Apiary II	Apiary III	Average
Antioxidant Activity DPPH [%] (*n* = 3) (±SD)					
7-day-old	water	51.0 ^d^ ± 0.4	41.9 ^b^ ± 1.6	57.4 ^f^ ± 0.9	50. ± 5.3
	ethanol 70%	20.5 ^F^ ± 0.1	13.9 ^E^ ± 2.6	10.4 ^C^ ± 1.6	14.9 ± 5.1
11-day-old	water	61.3 ^g^ ± 0.2	53.0 ^e^ ± 0.6	51.5 ^de^ ± 0.6	55.2 ± 7.8
	ethanol 70%	20.2 ^F^ ± 3.8	15.3 ^E^ ± 1.1	8.2 ^B^ ± 0.5	14.6 ± 6.0
14-day-old	water	57.2 ^f^ ± 1.1	44.5 ^c^ ± 1.0	37.4 ^a^ ± 0.4	43.3 ± 10.0
	ethanol 70%	6.3 ^A^ ± 1.3	15.4 ^E^ ± 1.1	13.0 ^DE^ ± 1.6	11.5 ± 4.7
Antioxidant Activity ABTS [%] (*n* = 3) (±SD)					
7-day-old	water	36.6 ^e^ ± 0.4	25.9 ^c^ ± 1.0	49.5 ^g^ ± 0.5	37.3 ± 11.9
	ethanol 70%	68.2 ^E^ ± 1.3	70.9 ^F^ ± 1.6	56.5 ^C^ ± 1.1	65.2 ± 7.7
11-day-old	water	6.3 ^a^ ± 3.7	19.9 ^b^ ± 0.5	28.8 ^d^ ± 0.4	18.3 ± 11.3
	ethanol 70%	64.3 ^D^ ± 0.3	71.3 ^F^ ± 0.4	44.9 ^B^ ± 0.4	60.1 ± 13.7
14-day-old	water	30.0 ^d^ ± 0.3	30.4 ^d^ ± 0.3	40.2 ^f^ ± 0.3	33.5 ± 5.8
	ethanol 70%	68.8 ^E^ ± 0.4	32.1 ^A^ ± 0.5	55.9 ^C^ ± 0.3	52.3 ± 18.6
Ferric Reducing Antioxidant Power FRAP [mmol/100 g] (*n* = 3) (±SD)					
7-day-old	water	3.0 ^d^ ± 0.2	2.2 ^a^ ± 0.1	20.9 ^d^ ± 0.1	2.7 ± 0.4
	ethanol 70%	0.8 ^C^ ± 0.1	0.7 ^BC^ ± 0.0	0.6 ^B^ ± 0.0	0.7 ± 0.1
11-day-old	water	3.2 ^e^ ± 0.2	2.6 ^c^ ± 0.1	3.4 ^f^ ± 0.2	3.0 ± 0.4
	ethanol 70%	1.0 ^D^ ± 0.0	0.7 ^BC^ ± 0.0	0.7 ^BC^ ± 0.9	0.8 ± 0.2
14-day-old	water	2.4 ^b^ ± 0.1	2.2 ^a^ ± 0.1	2.0 ^a^ ± 0.1	2.2 ± 0.2
	ethanol 70%	0.4 ^A^ ± 0.1	0.6 ^BC^ ± 0.0	0.4 ^A^ ± 0.0	0.5 ± 0.2
Total Phenolic Content TPC [mg GAE/100 g] (*n* = 3) (±SD)					
7-day-old	water	827.3 ^c^ ± 29.8	699.9 ^b^ ± 32.4	731.2 ^b^ ± 24.6	763.9 ± 66.4
	ethanol 70%	279.3 ^D^ ± 17.5	281.3 ^E^ ± 21.0	231.2 ^C^ ± 13.5	263.9 ± 28.4
11-day-old	water	1005.0 ^d^ ± 28.2	832.8 ^c^ ± 18.0	984.1 ^d^ ± 24.6	940.6 ± 94.0
	ethanol 70%	399.3 ^H^ ± 14.6	298.6 ^G^ ± 17.0	144.8 ^A^ ± 16.6	280.9 ± 128.2
14-day-old	water	640.9 ^a^ ± 91.2	811.0 ^c^ ± 20.3	829.4 ^c^ ± 14.0	760.4 ± 103.9
	ethanol 70%	200.4 ^B^ ± 18.1	290.2 ^F^ ± 21.0	284.7 ^E^ ± 18.6	258.4 ± 50.3
Total Flavonoid Content TFC [mg/100 g] (*n* = 3) (±SD)					
7-day-old	water	104.5 ^g^ ± 1.2	73.6 ^d^ ± 5.7	59.2 ^b^ ± 1.1	79.0 ± 23.2
	ethanol 70%	43.8 ^D^ ± 1.1	18.4 ^B^ ± 1.2	15.0 ^A^ ± 4.8	25.7 ± 15.7
11-day-old	water	122.0 ^h^ ± 1.1	72.4 ^d^ ± 3.0	87.5 ^f^ ± 2.2	94.0 ± 25.4
	ethanol 70%	49.8 ^E^ ± 5.1	54.0 ^F^ ± 3.8	17.6 ^B^ ± 0.5	40.5 ± 19.9
14- day-old	water	82.3 ^e^ ± 5.8	63.2 ^c^ ± 3.7	42.5 ^a^ ± 4.3	62.7 ± 19.9
	ethanol 70%	19.2 ^B^ ± 2.1	57.2 ^G^ ± 4.1	22.6 ^C^ ± 1.2	33.0 ± 21.0

^a, b, c, d, e, f, g, h^—for aqueous extracts lowercase superscript letters indicate significant differences for data in columns (apiary number) and rows (developmental stage) obtained by two-way ANOVA (NIR Fisher test. *p* < 0.05). ^A, B, C, D, E, F—^for ethanolic extracts uppercase superscript letters indicate significant differences for data in columns (apiary number) and rows (developmental stage) obtained by two-way ANOVA (NIR Fisher test. *p* < 0.05). SD is the standard deviation.

## Data Availability

Data is contained within the article or supplementary material.

## Supplementary Materials

The following are available online at https://www.mdpi.com/article/10.3390/antiox10050639/s1, Table S1: Correlation matrix for frozen and freeze-dried drone brood homogenate, Table S2: Characterization of chromatographic bands for reference standards, color and intensity of band, Table S3: Characterization of chromatographic bands for extracts of drone brood homogenates, color and intensity of band in UV_366_ and after derivatization.
