# Supplementary material for: Antioxidant Activity of Frozen and Freeze-Dried Drone Brood Homogenate Regarding the Stage of Larval Development
Source: Antioxidants (Basel). 2021 Apr 22;10(5):639. doi: 10.3390/antiox10050639 (PMC8143536; doi:10.3390/antiox10050639)
Supplement: Supplementary file 1 [file antioxidants-10-00639-s001.zip › antioxidants-1175841-supplementary.pdf]

Table S1. Correlation matrix for frozen and freeze-dried drone brood homogenate.

		DPPH		ABTS		FRAP		TPC		TFC	
		Frozen	Freeze-dried	Frozen	Freeze-dried	Frozen	Freeze-dried	Frozen	Freeze-dried	Frozen	Freeze-dried
DPPH	Frozen	1									
	Freeze-dried	0.824*	1								
ABTS	Frozen	-0.828	-0.760	1							
	Freeze-dried	-0.833	-0.828	0.833*	1						
FRAP	Frozen	0.914*	0.876*	-0.700	-0.714	1					
	Freeze-dried	0.865*	0.814*	-0.714	0.700*	0.909*	1				
TPC	Frozen	0.929*	0.892*	-0.840	-0.817	0.883*	0.919*	1			
	Freeze-dried	0.871*	0.929*	-0.762	0.840*	0.917*	0.883*	0.935*	1		
TFC	Frozen	0.838*	0.911*	-0.773	-0.734	0.882*	0.962*	0.883*	0.908*	1	
	Freeze-dried	0.823*	0.838*	-0.672	0.773*	0.717*	0.882*	0.749*	0.883*	0.799*	1

*—Correlation coefficient statistically significant ($p < 0.05$).

Table S2. Characterization of chromatographic bands for reference standards, color and intensity of band.

Standard	R_f	UV_{366} color	DPPH derivatization intensity
p-coumaric acid	0.41	blue	++
apigenin-7-glucoside	0.05	yellow	-
ferulic acid	0.42	blue	+++
naringenin	0.52	yellow	+++
apigenin	0.43	yellow	+++
chrysin	0.63	pale blue	++

Bands color: + slightly intense; ++ medium intensive; +++ very intense.

Table S3. Characterization of chromatographic bands for extracts of drone brood homogenates, color and intensity of band in UV_{366} and after derivatization.

**Band number**			**1**	**2**	**3**	**4**	**5**	**6**
R_f			0.09	0.22	0.30	0.33	0.45	0.80
UV_{366} color			blue	blue	pale yellow	pale blue	yellow	blue
UV_{366} intensity	I apiary	7- day-old	+++	++	+	+	++	++
		11- day-old	+++	+	+	+	++	+
		14- day-old	++	+	+	-	-	++
	II apiary	7- day-old	+++	++	++	+	++	++
		11- day-old	+++	++	++	+++	++	+
		14- day-old	+++	++	+++	+++	+++	++
	III apiary	7- day-old	+++	++	++	+++	++	+
		11- day-old	++	+	++	++	-	+
		14- day-old	++	+	++	++	-	+
DPPH derivatization intesity	I apiary	7- day-old	++	+	++	-	++	-
		11- day-old	++	+	++	-	+	-
		14- day-old	++	+	+++	-	+	-
	II apiary	7- day-old	+++	+	+	++	+++	++
		11- day-old	++	+	+	++	+++	+
		14- day-old	++	+	+	+++	+++	+
	III apiary	7- day-old	+++	+	+	+++	+++	++
		11- day-old	++	+	+	+	++	+
		14- day-old	++	+	+++	+	+	-
Identification			phenolic acid	phenolic acid	flavonoid	?	**apigenin**	?

Bands color: + slightly intense; ++ medium intensive; +++ very intense.
